# Differential expression of BCL11b and CDKN2A in CD30-positive peripheral T cell lymphoma: Retrospective study

**DOI:** 10.1097/MD.0000000000035531

**Published:** 2023-11-17

**Authors:** Yajing Wang, Fei Zhang, Ning Gao, Peng Bu, Wei Cui, Yanfeng Xi

**Affiliations:** a Department of Pathology, Shanxi Province Cancer Hospital/ Shanxi Hospital Affiliated to Cancer Hospital, Chinese Academy of Medical Sciences/Cancer Hospital Affiliated to Shanxi Medical University, Taiyuan, People’s Republic of China.

**Keywords:** BCL11b, CD30, CDKN2A, peripheral T cell lymphoma

## Abstract

Peripheral T-cell lymphoma is a disease that includes multiple T-cell lymphoma subtypes. It is still unclear whether CD30 can be used as a new target molecule and classification standard for PTCL. Differences in the molecular characteristics of CD30-positive PTCL and CD30-negative PTCL have rarely been reported. This study aimed to analyze the expression of BCL11b and CDKN2A in CD30-positive PTCL and CD30-negative PTCL, in order to guide the pathological classification, prognosis, and clinical treatment of PTCL. Immunohistochemical staining and quantitative reverse-transcription PCR (qRT-PCR) were performed on formalin-fixed paraffin-embedded tissue. Verification of BCL11b and CDKN2A expression in ALCL, PTCL-NOS, AITL and NK/TCL. Based on immunohistochemical analysis, the expression level of BCL11b in the lymph node reactive hyperplasia control group was high at 85.0%, which was higher than 68.8% in CD30-positive PTCL and 44.1% in CD30-negative PTCL (*P* < .05, respectively). CDKN2A showed expression rates of 70.0% in the control group, 79.2% in CD30-positive PTCL and 79.4% in CD30-negative PTCL. qRT-PCR showed that the relative BCL11b mRNA expression levels in patients with PTCL were lower than those in the control group (0.694 vs 1.832, *P* = .045). Univariate analysis showed that international prognostic index score, CD30 expression, and BCL11b expression were closely related to prognosis (*P* < .05, respectively). Multivariate Cox regression analysis revealed that high expression of BCL11b mRNA was an independent factor affecting prognosis (respectively, *P* < .05). Spearman correlation analysis indicated that BCL11b expression had a significant positive correlation with CD30 expression (*P* = .005). These results indicate that BCL11b may be involved in CD30 differentiation and PTCL prognosis. The detection and targeting of BCL11b and CD30 may provide new strategies for the treatment and classification of PTCL.

## 1. Introduction

Peripheral T-cell lymphoma (PTCL) are tumors originating from mature T lymphocytes, and account for 10% to 15% of non-Hodgkin lymphoma.^[1]^ Patients with PTCL have worse prognoses than aggressive B-cell lymphoma patients.^[2]^ Peripheral T-cell lymphoma- not otherwise specified (PTCL-NOS) accounts for a high proportion of all PTCL, but lacks clear diagnostic criteria and treatment due to the heterogeneity of immunophenotype and unclear pathological characteristics. The overall survival (OS) and failure-free survival (FFS) of 10–15 years are only 10%.^[3]^

The standard “CHOP” (cyclophosphamide, adriamycin, vincristine, prednisone) therapy regimen is not ideal for most patients with PTCL, and the 5-year progression-free survival is only 13% to 36%,^[2]^ and recurrence and inability to alleviate is still the main problem in the treatment. The median progression-free survival of recurrent/ refractory PTCL ranged from 1.8 months to 4 months.^[4]^ Three drugs(pralatrexate, romidepsin, belinostat) approved by FDA for the treatment of PTCL lack effective biomarkers. Recently, a new antibody conjugate targeting CD30, Brentuximab (BV), has been applied to the treatment of anaplastic large cell lymphoma (ALCL) in PTCL. The BV can reduce the tumor size of some patients and prolong the 5 year overall survival rate, showing an encouraging effect.^[5]^

CD30 is a member of the tumor necrosis factor receptor superfamily, which regulates lymphocyte proliferation and differentiation. The expression of CD30 is limited in normal or inflammatory tissues. Whereas in lymphocytic proliferative diseases, CD30 is mainly expressed by diseased lymphocytes in classical Hodgkin lymphoma, ALCL, and other PTCLs to varying degrees.^[6]^ CD30 is a tumor marker and can also be used for auxiliary diagnosis of lymphoma subtypes, and is a valuable biomarker for precise lymphoma treatment. However, differences in the molecular characteristics of CD30-positive PTCL and CD30-negative PTCL have rarely been reported. Whether CD30 expression and signal pathway lead to cell cycle arrest, apoptosis and even proliferation of CD30 positive lymph proliferation remains controversial.

Transcription factors are a class of protein molecules that bind to specific DNA sequences to ensure that target genes are expressed at a specific intensity in a specific time and space, and their expression changes are an important pathological basis for malignant tumors. However, with the deepening understanding of the role of transcription factors in diseases, these transcription factors are considered promising therapeutic targets for various malignant tumors.

In recent studies, PTCL-NOS can be divided into GATA3 and TBX21 subgroups according to gene expression profiles.^[7]^ Heavican et al^[8]^ found that the expression of B-cell CLL/lymphoma 11b (BCL11b) increased in the PTCL-TBX21 group with better prognosis, whereas in the PTCL-GATA3 group, cyclin-dependent kinase inhibitor 2A (CDKN2A) showed that gene deletion reduced mRNA expression.

BCL11b, also known as Rit1 and CTIP2, is a newly discovered C2H2 transcription factor in the B-cell lymphoma/leukemia gene family. It is located at 14q32.31 of the long arm of chromosome 14, and encodes a zinc finger protein expressed in T cells, but not in B cells. It is expressed in T cells, thymocytes, neurons, mitogen proteins, teeth, and other tissues.^[9]^ BCL11b could inhibit cyclin - dependent kinase inhibitors, like p21/Cip2/Waf1 and p57/Kip2.^[10]^ In the thymus of animals lacking BCL11b, the development of CD4/CD8 double-negative cells is blocked. So, BCL11b is one of the genes necessary for T cell differentiation and survival.

CDKN2A is a negative regulator and tumor suppressor gene at the checkpoint of the cell cycle G1/S phase,^[11]^ located on chromosome 9p21. The CDKN2A protein encoded by CDKN2A (also known as P16INK4a) is a key negative regulator of cell cycle regulation. It inhibits phosphorylation of Rb and CDK7, inducing G1/S phase arrest, and inhibits RNA polymerase II carboxyl end and JNK1/3 kinase activity. In addition, abnormal expression of the CDKN2A gene can cause genomic instability. CDKN2A deletion is a common mutation in T-cell acute lymphoblastic leukemia.^[12]^

In this study, BCL11b and CDKN2A expression in CD30-positive PTCL was studied via immunohistochemistry and RT-PCR, to explore the effect of transcription factors and cell cycle regulators involved in T cell proliferation and differentiation on CD30 surface antigen expression, and the molecular relationship between CD30-positive PTCL and CD30-negative PTCL, in order to guide the pathological classification, prognosis, and clinical treatment of PTCL.

## 2. Materials and methods

### 2.1. Sample collection

We collected paraffin-embedded specimens of patients diagnosed with PTCL from January 2010 to June 2020 in the Department of Pathology, Shanxi Cancer Hospital, and obtained clinical follow-up data from 82 patients. All patients agreed to be followed up for sampling. And all cases had complete clinical course records. Case inclusion criteria: All patients were primary disease, had no previous history of tumor, and had not received radiotherapy or chemotherapy before surgery. All cases were confirmed by immunohistochemistry: CD20, CD79a, CD3, CD5, CD4, CD8, CD21, CD23, BCL2, BCL6, CD10, CyclinD1, CXCL-13, EBER, C-myc, CD56, Granzyme B, ALK, Ki-67.

Among them, 48 cases were CD30-positive, including PTCL, 12 cases were peripheral T-cell lymphoma, not otherwise specified (PTCL-NOS), 14 cases were angioimmunoblastic T-cell lymphoma (AITL), 5 cases of NK/T-cell lymphoma (NK/TCL), and 17 cases were anaplastic large cell lymphoma (ALCL) (ALK + ALCL 8 cases, ALK-ALCL 9 cases). CD30-negative PTCL 34 cases included PTCL-NOS in 21 cases, AITL in 9 cases, and NK/TCL in 4 cases. Follow-up time to death or end of follow-up. The selected patients were not treated with radiotherapy or chemotherapy before surgery. For patients with ALCL, most of them were treated with BV Vedotin after surgery. For the other patients with PTCL, most of them were treated with CHOP-E (Etoposide) regimen. Support therapy was used in a very small part. Another 20 cases of lymph node reactive hyperplasia were selected as controls. The use of materials and clinical information was approved by the Research Ethics Committee of Shanxi Cancer Hospital.

### 2.2. Immunohistochemistry

Immunohistochemical detection was performed on formalin-fixed paraffin-embedded lymph node specimens using a Roche Benchmark®XT automatic staining machine (Roche, Switzerland). Rabbit anti-human BCL11b monoclonal antibody was purchased from Novus Biologicals (NB100-2600) according to 1: 1600 the configuration antibody working fluid. Rabbit anti-human CDKN2A monoclonal antibody was purchased from Abcam (ab3642) and diluted 1: 80 dilution to configure the antibody working fluid. Mouse anti-human CD30 monoclonal antibody was purchased from Gene Technology Co., Ltd. (JCM182), which was used as the working fluid.

Positive BCL11b is located in the nucleus. Five high power fields were randomly selected for each case (× 400), according to the percentage of positive cells and staining intensity score: the percentage of positive cells score: no positive cells or positive expression rate ≤19% for 0, 20% to 39% for 1, 40% to 59% for 2, 60% to 79% for 3, 80% to 100% for 4. Dyeing intensity score: 0, colorless, 1 light yellow, 2 brown yellow; and 3, dark brown. The 2 values were multiplied as the final score. 0 to 3 points (−), 4–5 points (+), 6–7 points (+ +), and ≥ 8 points (+ + +). The positive expression rate was calculated.^[13]^ CDKN2A protein was accumulated in the cytoplasm and nucleus, and immunohistochemical staining results were performed according to the interpretation standard of cytoplasmic/nuclear molecular: 0 (negative): the target cells were not stained, 1 + (weakly positive): ≤ 10% cytoplasm/nucleus showed different degrees of brown yellow particles., 2 + (positive): 10% to 30% of the cytoplasm/nucleus showed moderate staining, or ≤ 70% of the cytoplasm/nucleus showed weak or moderate staining, 3 + (strong positive): > 30% of the cytoplasm/nucleus showed strong positive staining, and > 70% of the cytoplasm/nucleus showed moderate positive staining.^[14]^

The positive expression of CD30 protein was localized in the membrane. The percentage of tumor cells with positive staining above moderate intensity was evaluated, and the positive expression of >15%^[15]^ tumor cells was defined as CD30-positive PTCL; otherwise, it was CD30-negative PTCL.

### 2.3. Isolation of total RNA and reverse transcription

Total RNA was extracted from formalin-fixed paraffin-embedded specimens using a total RNA extraction kit (AmoyDx, China) according to the manufacturer instructions. The first strand cDNA synthesis kit (Thermo Science TM Fermentas) was used to reverse transcribe RNA according to the manufacturer's instructions.

### 2.4. Quantitative reverse transcription-PCR (qRT-PCR)

To quantify the mRNA expression of BCL11b and CDKN2A, according to manufacturer instructions, real-time RT-PCR was performed in 20 μL reaction volume using Fast SYBR ® Green Master Mix (Applied Biosystems). All reactions were performed on a Step ONE^TM^ amplification system (Applied Biosystems), and the *GAPDH* gene was used as an endogenous control.

The primer sequences of BCL11b gene were as follows: forward 5’-TCCAGCTACATTTGCACAACA-3 and reverse 5’-GCTCCAGGTAGATGCGGAAG-3’. CDKN2A gene primer sequence: forward 5’-CAAGATCACGCAAACCTCTG-3’ and reverse 5’-CGACCCTATACACGTTGAACTG-3’. The GAPDH sequence was as follows: forward 5’-CTCACCGGATGCACCAATGTT-3’ and reverse 5’-CGCGTTGCTCAATGTTCAT-3’. Thermal cycles of 40 cycles (3 seconds) and 30 seconds (60 °C) were performed at 95 °C and 60 °C, respectively. The initial denaturation step was 95 °C for 20 seconds.

The gene expression value was normalized to the endogenous control *GAPDH* and calibrated to the sample with the lowest expression level. The relative quantification (RQ) was calculated using the 2 − ΔΔCt method proposed by Livak, RQ = 2 − ^ΔΔCt,^ (ΔΔCt = ΔCt − ΔCt ^control,^ where ΔCt = Ct ^target gene^ − Ct^GAPDH^).

### 2.5. Statistical analysis

Statistical analysis was performed using SPSS 25.0. The positive expression rates of BCL11b and CDKN2A proteins were compared between groups using the χ^2^ test, and the differences in mRNA levels were compared using the Mann–Whitney U test. The Spearman rank correlation coefficient was used to calculate the correlation between the variables. The overall survival rate of PTCL was tested using the Kaplan–Meier method, and the difference was tested by log-rank (GraphPad Prism software). Multivariate Cox proportional hazard regression analysis was used to evaluate independent prognostic factors related to patient survival. Statistical significance was set at *P* < .05.

## 3. Results

### 3.1. Immunohistochemistry results

#### 3.1.1. Expression of BCL11b.

**Figure 1.**
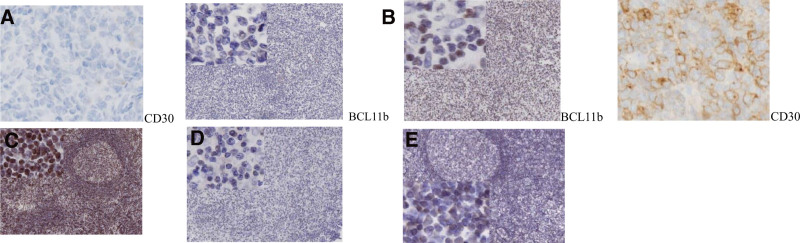
BCL11b and CDKN2A expression was detected by immunohistochemistry. (A) BCL11b is negative in CD30-negative PTCL. (B) BCL11b is weakly positive in CD30-positive PTCL. (C) BCL11b is strongly expressed in lymph node reactive hyperplasia tissues. (D) CDKN2A is negatively expressed in PTCL. (E) CDKN2A is moderately positive in lymph node reactive hyperplasia. BCL11b = B-cell CLL/lymphoma 11b, CDKN2A = cyclin-dependent kinase inhibitor 2A.

In PTCL tissue, the positive rate of BCL11b protein was 58.5% (48/82, shown in Fig. 1). The BCL11b protein positivity rate was 85.0% (17/20) in 20 cases of lymph node reactive hyperplasia, and the difference was statistically significant (χ^2^ = 4.871, *P* = .027). In CD30-positive PTCL tissue, the high expression rate of BCL11b was 68.8% (33/48), which was higher than 44.1% (15/34) in CD30-negative PTCL tissue (χ^2^ = 4.975, *P* = .026), shown in Table 1 and Figure 1.

**Table 1:** Positive rates of BCL11b and CDKN2A in CD30 +/CD30-PTCL.

	BCL11b positive rate			CDKN2A positive rate		
	CD30-positive rate (%)	CD30-negative rate (%)	*P* value	CD30-positive rate (%)	CD30-negative rate (%)	*P* value
Total	68.8%	44.1%	.026*	79.2%	79.4%	.978
PTCL-NOS	83.3%	47.6%	.043*	100.0%	71.4%	.443
AITL	57.1%	33.3%	.265	100.0%	100.0%	/
NK/TCL	80.0%	50.0%	.343	100.0%	75.0%	.063
ALCL	64.7%	/	/	82.4%	/	/

AITL = angioimmunoblastic T-cell lymphoma, ALCL = anaplastic large cell lymphoma, BCL11b = B-cell CLL/lymphoma 11b, CDKN2A = cyclin-dependent kinase inhibitor 2A, NK/TCL = NK/T-cell lymphoma, PTCL-NOS = Peripheral T-cell lymphoma- not otherwise specified.

#### 3.1.2. Expression of CDKN2A.

In PTCL tissue, the positive rate of CDKN2A protein was 79.3% (65/82, shown in Fig. 1). In 20 cases of lymph node reactive hyperplasia tissue specimens, the positive rate of CDKN2A protein was 70.0% (14/20), and the difference was not statistically significant (*P* > .05). In CD30-positive PTCL tissue, the positive expression rate of CDKN2A was 79.2% (38/48), which was not significantly different from 79.4% (27/34) in CD30-negative PTCL tissue (*P* = .978), shown in Table 1 and Figure 1.

### 3.2. Relationship between the expression of BCL11b and clinicopathological factors of PTCL

There was no correlation between the expression of BCL11b protein and BCL11b mRNA and clinicopathological factors, and there was no difference in the expression of BCL11b protein and BCL11b mRNA in 4 different pathological types of PTCL, shown in Table 2.

**Table 2:** Relationship between the expression of BCL11b and clinicopathological factors of PTCL.

Clinical characteristics	Cases	BCL11b protein expression			BCL11b mRNA	
		-	+	*P* -value	relative expression	*P* value
Age				.913		.791
<60 (46.27 + 13.79)	44	18	26		0.585	
≥60 (69.03 + 6.88)	38	16	22		0.746	
Sex				.794		.363
Male	52	21	31		0.746	
Female	30	13	17		0.359	
B symptom				.290		.243
Yes	33	16	17		0.818	
No	49	18	31		0.423	
LDH				.099		.298
High	37	19	18		0.847	
Normal	45	15	30		0.400	
Ann Arbor stage				.069		.501
I–II	26	7	19		0.807	
III–IV	56	27	29		0.464	
IPI				.260		.729
0–2	47	17	30		0.690	
3–4	35	17	18		0.697	
Pathological type				.793		.406
PTCL-NOS	33	13	20		0.480	
AITL	23	12	11		0.865	
NK/TCL	9	3	6		0.252	
ALK + ALCL	8	3	5		0.870	
ALK-ALCL	9	3	6		0.818	

AITL = angioimmunoblastic T-cell lymphoma, ALCL = anaplastic large cell lymphoma, BCL11b = B-cell CLL/lymphoma 11b, IPI = international prognostic index, LDH = layered double hydroxide, NK/TCL = NK/T-cell lymphoma, PTCL-NOS = Peripheral T-cell lymphoma- not otherwise specified.

### 3.3. Expression of BCL11b, CDKN2A by qRT-PCR

**Figure 2.**
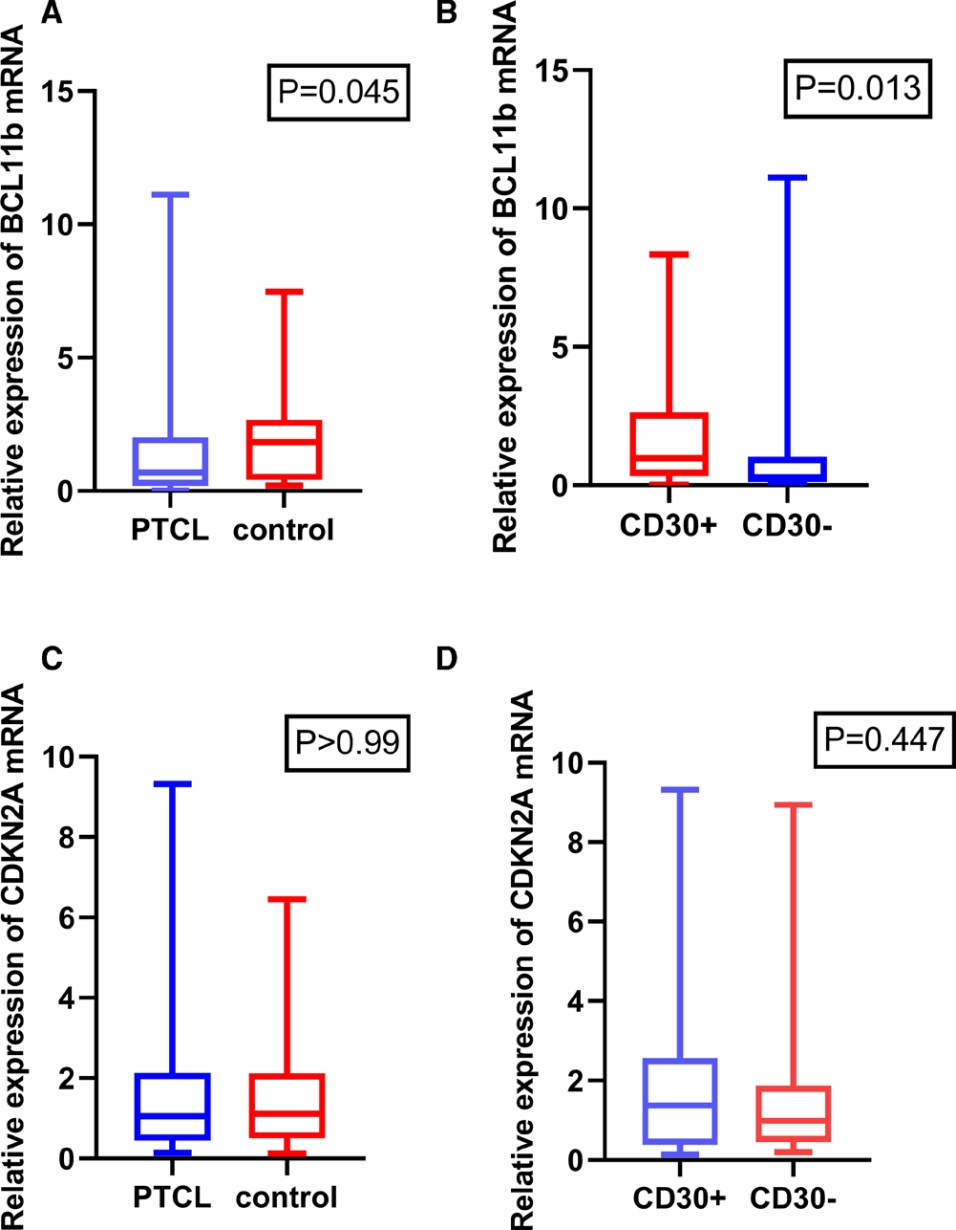
Quantitative RT-PCR results showed that the relative expression of BCL11b mRNA in PTCL patients was lower than that in the control group (0.694 vs 1.832, *P* = .045, Fig. 1A), and the relative expression of BCL11b mRNA in CD30-PTCL patients was lower than that in CD30 + PTCL patients (0.293 vs 0.984, *P* = .013, Fig. 1B). There was no significant difference in the relative expression of CDKN2A mRNA among patients with reactive proliferative lymph nodes, CD30 + PTCL and CD30- PTCL (1.113 vs 1.054 and 1.371 vs 0.988, *P* > .05, Fig. 1C and D). BCL11b = B-cell CLL/lymphoma 11b, CDKN2A = cyclin-dependent kinase inhibitor 2A.

BCL11b mRNA and CDKN2A mRNA expression in reactive proliferative lymph nodes, CD30-positive PTCL, and CD30-negative PTCL were compared, shown in Figure 2.

### 3.4. Survival analysis

**Figure 3.**
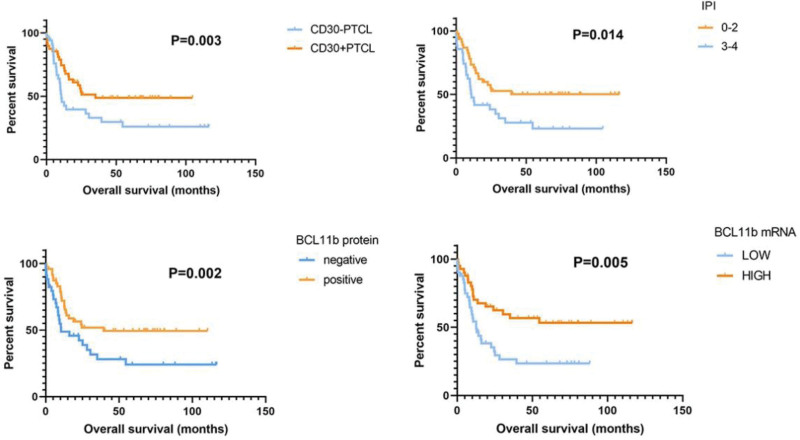
Overall survival (OS) analysis for PTCL patients.

As of June 2020, the median follow-up time of 82 patients was 19.63 months (from 0.13 months, 115.97 months). The Kaplan–Meier method was used for survival data analysis, and the log-rank test was used for survival curve plotting. BCL11b and CDKN2A mRNA were divided into high expression and low expression groups according to the median. We found that compared with CD30-negative PTCL, CD30-positive PTCL patients had a higher overall survival rate and better prognosis (*P* = .003). The median survival time of patients with an international prognostic index score of 0 to 2 was 24.7 months, higher than 10.7 months of patients with an international prognostic index score of 3 to 4 (*P* = .014). The overall survival rate of PTCL patients with high BCL11b gene expression was significantly higher than that of PTCL patients with low BCL11b gene expression (*P* = .002) (Fig. 3).

Cox regression analysis revealed that BCL11b mRNA expression was an independent prognostic factor (*P* = .005). In patients with high BCL11b mRNA expression, the risk of death was 59.6% lower than that in patients with low BCL11b mRNA expression, shown in Table 3.

**Table 3:** Cox multivariate regression analysis of BCL11b expression and related factors.

Related factors	B	SE	WALD	*P* value	HR	95%CI
IPI	0.566	0.311	3.312	.069	1.762	0.957–3.243
BCL11b protein	−0.519	0.318	2.668	.102	0.595	0.319–1.109
BCL11b mRNA	−0.906	0.319	8.069	.005*	0.404	0.216–0.755
CD30	−0.167	0.320	0.271	.603	0.847	0.452–1.585
LDH	−0.221	0.325	0.464	.496	1.247	0.660–2.357
SEX	−0.040	0.315	0.016	.899	1.041	0.561–1.930

BCL11b = B-cell CLL/lymphoma 11b, IPI = international prognostic index, LDH = layered double hydroxide.

### 3.5. Correlation study on the expression of BCL11b, CDKN2A, and CD30

Spearman correlation analysis showed that BCL11b expression was significantly positively correlated with CD30 expression (rs = 0.490, *P* = .005).

## 4. Discussion

Peripheral T-cell lymphoma is a rare and highly heterogeneous group of tumor, originating from mature T cells. The molecular phenotype and pathogenesis of peripheral T-cell lymphoma remain unclear, and there is a lack of specific therapeutic targets and prognostic markers. Studying the mechanism of PTCL at the molecular level helps find new molecular targets and find new treatment methods to reduce pain and prolong the survival time of patients.

In this study, PTCL samples were divided into 2 groups according to the expression of CD30 protein in the clinicopathological data. The results showed that CD30-positive PTCL showed better clinical results than CD30-negative PTCL, which was consistent with the findings of Bisig,^[16]^ suggesting that the expression of CD30 protein may divide PTCL into 2 different subgroups. In addition, the expression of BCL11b in PTCL and normal tissues, CD30-positive PTCL and CD30-negative PTCL were different, and BCL11b protein was an independent prognostic factor of the disease. Therefore, we speculated that BCL11b might be used to guide the classification and prognosis of PTCL.

However, there was no statistically significant difference in the expression of CDKN2A between PTCL and normal tissues and CD30-positive PTCL and CD30-negative PTCL, which was inconsistent with the conclusion of Maura,^[17]^ who suggested that the lack of CDKN2A is related to the poor prognosis of PTCL-NOS. A possible reason is that the expression of CDKN2A is different in different pathological types of PTCL, and the interpretation of immunohistochemical results may lack relative objectivity, which needs further exploration.

Correlation analysis showed a positive correlation between BCL11b protein expression and CD30 expression, and there was no correlation between CDKN2A protein expression and CD30 expression. This result suggests that transcription factor BCL11b may affect CD30 expression, and BCL11b may be involved in CD30 signaling pathway, leading to abnormal cell cycle in lymph node proliferation. While the transcription factor CDKN2A involved in cell cycle G1/S phase regulation does not affect CD30 expression. It was found that^[18]^ the expression of BCL11b was induced at the early stage of T cell differentiation in the human thymus. The up-regulation of BCL11b was closely related to the T-spectrum typing and the subsequent differentiation into CD4 + CD8 + double-positive cells. Therefore, we can conclude that BCL11b may be involved in the differentiation of surface antigen CD30 during T cell development, and thus affect the classification and prognosis of PTCL.

Insufficient expression of BCL11b damages T cell typing and induces differentiation stagnation at the initial stage of human T cell differentiation. In Chiarle, R.,^[19]^ CD30 overexpression resulted in losing CD4 + CD8 + thymocytes. Therefore, the effect of BCL11b on CD30 expression and whether BCL11b can be used as another therapeutic target for lymphoma may become a new research direction in the future.

**Figure 4.**
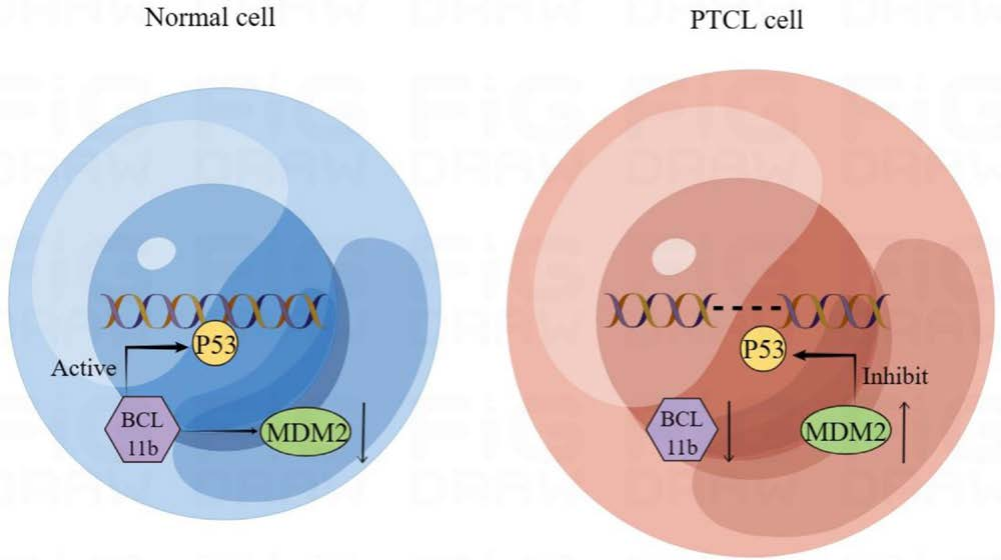
In normal cell, BCL11b inhibit the expression of MDM2 and activate p53 to complete DNA repair when DNA damage occurs. In PTCL cell, decreased BCL11b expression leads to enhanced MDM2 function, which in turn inhibits p53 repair of DNA. BCL11b = B-cell CLL/lymphoma 11b.

Our study found that low expression of BCL11b in PTCL often indicates a poor prognosis. Compared with CD30-positive PTCL, CD30-negative PTCL with lower expression of BCL11b showed worse clinical prognosis, which may be related to MDM2. MDM2 is a negative regulator of the tumor suppressor p53, which can inhibit p53 as a guardian of the genome to repair damaged DNA double strands, thereby damaging the stability and integrity of genes. Studies^[20]^ have shown that BCL11b can inhibit the expression of MDM2 and activate p53 to complete DNA repair when DNA damage occurs. Therefore, the expression of BCL11b and p53 is synergistic (shown in Fig. 4).

It can be inferred that the expression of BCL11b is downregulated in PTCL, and the enhancement of MDM2 function causes a decrease in p53 expression and a decrease in cell cycle inhibitor and pro-apoptotic protein of p53 transcription. Therefore, we speculated that the decreased expression of BCL11b may promote the clinical malignant behavior of peripheral T-cell lymphoma through this mechanism.

In summary, we found that BCL11b protein is an independent prognostic factor for PTCL, and BCL11b protein is highly correlated with CD30 expression, which further suggests that BCL11b and CD30 are involved in the progression and prognosis of PTCL. However, our study is only limited to the verification of protein and mRNA level, lacks the verification of cell experiments and in vitro experiments. and the relevant mechanism of BCL11b in PTCL still needs to be further explored. We speculate that enhanced expression of BCL11b carried out simultaneously with anti-CD30 targeted drugs, which may bring greater benefits to PTCL patients.

## Acknowledgments

The authors would like to thank you to all those who work in Department of Pathology, Shanxi Cancer Hospital. for their support.

## Author contributions

**Methodology:** Ning Gao, Peng Bu.

**Writing – original draft:** Yajing Wang.

**Writing – review & editing:** Fei Zhang, Wei Cui, Yanfeng Xi.

## References

[1] Rüdiger T Weisenburger DD Anderson JR. Non-Hodgkin's Lymphoma Classification Project. Peripheral T-cell lymphoma (excluding anaplastic large-cell lymphoma): results from the Non-Hodgkin’s Lymphoma Classification Project. Ann Oncol. 2002;13:140–9. 11863096 10.1093/annonc/mdf033

[2] Khan N Ozkaya N Moskowitz A. Peripheral T-cell lymphoma - are we making progress? Best Pract Res Clin Haematol. 2018;31:306–14. 30213401 10.1016/j.beha.2018.07.010 PMC8941989

[3] Foss FM Zinzani PL Vose JM. Peripheral T-cell lymphoma. Blood. 2011;117:6756–67. 21493798 10.1182/blood-2010-05-231548

[4] Ng SY Jacobsen ED. Peripheral T-Cell lymphoma: moving toward targeted therapies. Hematol Oncol Clin North Am. 2019;33:657–68. 31229161 10.1016/j.hoc.2019.04.002

[5] Gualberto A. Brentuximab Vedotin (SGN-35), an antibody-drug conjugate for the treatment of CD30-positive malignancies. Expert Opin Investig Drugs. 2012;21:205–16. 10.1517/13543784.2011.641532 22127011

[6] Pierce JMR Mehta A. Diagnostic, prognostic and therapeutic role of CD30 in lymphoma. Expert Rev Hematol. 2017;10:29–37. 27927047 10.1080/17474086.2017.1270202

[7] Amador C Greiner TC Heavican TB. Reproducing the molecular subclassification of peripheral T-cell lymphoma-NOS by immunohistochemistry. Blood. 2019;134:2159–70. 31562134 10.1182/blood.2019000779 PMC6908831

[8] Heavican TB Bouska A Yu J. Genetic drivers of oncogenic pathways in molecular subgroups of peripheral T-cell lymphoma. Blood. 2019;133:1664–76. 30782609 10.1182/blood-2018-09-872549 PMC6460420

[9] Fang D Cui K Hu G. Bcl11b, a novel GATA3-interacting protein, suppresses Th1 while limiting Th2 cell differentiation. J Exp Med. 2018;215:1449–62. 29514917 10.1084/jem.20171127 PMC5940260

[10] Kominami R. Role of the transcription factor Bcl11b in development and lymphomagenesis. Proc Jpn Acad Ser B Phys Biol Sci. 2012;88:72–87. 10.2183/pjab.88.72 PMC3365246 22450536

[11] Savone D Carrone A Riganelli L. Management of HPV-related cervical disease: role of p16INK4a immunochemistry review of the literature. Tumori. 2016;102:450–8. 27443891 10.5301/tj.5000524

[12] Van Vlierberghe P Ferrando A. The molecular basis of T cell acute lymphoblastic leukemia. J Clin Invest. 2012;122:3398–406. 23023710 10.1172/JCI61269 PMC3461904

[13] Orth MF Hölting TLB Dallmayer M. High specificity of BCL11B and GLG1 for EWSR1-FLI1 and EWSR1-ERG positive Ewing Sarcoma. Cancers (Basel). 2020;12:644. 32164354 10.3390/cancers12030644 PMC7139395

[14] Horwitz SM Advani RH Bartlett NL. Objective responses in relapsed T-cell lymphomas with single-agent brentuximab vedotin. Blood. 2014;123:3095–100. 24652992 10.1182/blood-2013-12-542142 PMC4425442

[15] Lim AM Do H Young RJ. Differential mechanisms of CDKN2A (p16) alteration in oral tongue squamous cell carcinomas and correlation with patient outcome. Int J Cancer. 2014;135:887–95. 24436120 10.1002/ijc.28727

[16] Bisig B De Reyniès A Bonnet C. CD30-positive peripheral T-cell lymphomas share molecular and phenotypic features. Haematologica. 2013;98:1250–8. 23716562 10.3324/haematol.2012.081935 PMC3729906

[17] Maura F Dodero A Carniti C. <i>CDKN2A</i> deletion is a frequent event associated with poor outcome in patients with peripheral T-cell lymphoma not otherwise specified (PTCL-NOS). Haematologica. 2021;106:2918–26. 33054126 10.3324/haematol.2020.262659 PMC8561277

[18] Ha VL Luong A Li F. The T-ALL related gene BCL11B regulates the initial stages of human T-cell differentiation. Leukemia. 2017;31:2503–14. 28232744 10.1038/leu.2017.70 PMC5599326

[19] Chiarle R Podda A Prolla G. CD30 overexpression enhances negative selection in the thymus and mediates programmed cell death via a Bcl-2-sensitive pathway. J Immunol. 1999;163:194–205. 10384116

[20] Obata M Kominami R Mishima Y. BCL11B tumor suppressor inhibits HDM2 expression in a p53-dependent manner. Cell Signal. 2012;24:1047–52. 22245141 10.1016/j.cellsig.2011.12.026

